# Thermal decomposition of fullerene nanowhiskers protected by amorphous carbon mask

**DOI:** 10.1038/srep38760

**Published:** 2016-12-19

**Authors:** Hongxuan Guo, Chengxiang Wang, Kun’ichi Miyazawa, Hongxin Wang, Hideki Masuda, Daisuke Fujita

**Affiliations:** 1 Global Research center for Environment and Energy based on Nanomaterials Science (GREEN), National Institute for Materials Science (NIMS), 1-2-1 Sengen, Tsukuba, Ibaraki 305-0047, Japan; 2 Center for Nanoscale Science and Technology (CNST), National Institute of Standard and Technology (NIST), 100 Bureau Drive, Gaithersburg, MD 20899-6204, USA; 3 School of Material Science and Engineering, Shandong University, Jingshi Road No. 17923, Jinan 250061, China; 4 Fullerene Engineering Group, National Institute for Materials Science (NIMS), 1-1 Namiki, Tsukuba, Ibaraki 305-0044, Japan; 5 Advanced Key Technologies Division, Nano Characterization Unit, Surface Characterization Group, National Institute for Materials Science (NIMS), 1-2-1 Sengen, Tsukuba, Ibaraki 305-0047, Japan

## Abstract

Fullerene nanostructures are well known for their unique morphology, physical and mechanical properties. The thermal stability of fullerene nanostructures, such as their sublimation at high temperature is also very important for studying their structures and applications. In this work, We observed fullerene nanowhiskers (FNWs) *in situ* with scanning helium ion microscopy (HIM) at elevated temperatures. The FNWs exhibited different stabilities with different thermal histories during the observation. The pristine FNWs were decomposed at the temperatures higher than 300 °C in a vacuum environment. Other FNWs were protected from decomposition with an amorphous carbon (*a*C) film deposited on the surface. Based on high spacial resolution, *a*C film with periodic structure was deposited by helium ion beam induced deposition (IBID) on the surface of FNWs. Annealed at the high temperature, the fullerene molecules were selectively sublimated from the FNWs. The periodic structure was formed on the surface of FNWs and observed by HIM. Monte Carlo simulation and Raman characterization proved that the morphology of the FNWs was changed by helium IBID at high temperature. This work provides a new method of fabricating artificial structure on the surface of FNWs with periodic *a*C film as a mask.

In the last century, four types of allotropes of carbon, three-dimensional (diamond and graphite), two-dimensional (graphene), one-dimensional (nanotubes), and zero-dimensional (fullerenes) have been extensively studied. A zero-dimensional fullerene is a small spherical cluster created with 60 or more carbon atoms such as C_60_, C_70_, and so on^1^. From low dimensional to high dimensional, the fullerene molecules form various macro- and nano- crystalline structures *via* van der Waals bonds, such as fullerene nanotubes (FNTs) and fullerene nanowhiskers (FNWs)^2^,^3^. One dimensional fullerene nanostructures, especially FNTs and FNWs, are attractive for research since they can be used as FETs^4^,^5^, templates for nanostructures^6^, and others. Due to the low van der Waals bonds energy, FNWs are not stable at high temperature or in high energy electron irradiation^7^,^8^,^9^,^10^. As a crystalline structure made of fullerene with size of 1 nm, fullerene nano structure can be manipulated by a top-down nano fabrication in high spacial resolution^11^,^12^,^13^,^14^. However, to the knowledge of the author, artificial structure made on the surface of FNWs by a top down method have not been well investigated.

Different from other top-down nano fabrication methods, electron beam induced deposition (EBID) and ion beam induced deposition (IBID) are fast and resistless deposition techniques which can overcome the fundamental resolution limits of conventional lithography techniques^15^,^16^,^17^. Various materials can be deposited by using a scanning electron microscope (SEM) *via* EBID or a focused ion beam (FIB) system *via* IBID. For example, Pt, W and other materials have been deposited by using a gallium ion source FIB system with a gas injection system (GIS)^18^. Scanning helium ion microscopy (HIM) involves using an advanced scanning microscope with the same mechanism but with a higher resolution and greater focal depth than those with SEM and FIB systems^19^,^20^,^21^,^22^. Different materials, such as Pt, PtC, and W were deposited with C_9_H_16_Pt, (CH_3_)_3_Pt(C_P_CH_3_), and WF_6_ as precursors respectively, by using a HIM system with GIS^23^,^24^,^25^. Without GIS, amorphous carbon (*a*C) can also be deposited by EBID in electron microscope with hydrocarbon as a precursor^15^,^26^. In this process, the hydrocarbon absorbed on the surface of the samples decomposes into carbon atoms and is deposited again as *a*C film when the electron or ion beams scan the surface of the samples.

In this paper, we investigated the thermal decomposition of the FNW with an *in situ* heating sample holder setting in the HIM. During the scanning by helium ion beam, ion beam induced carbon deposited on the surface of FNW and formed a mask. A periodic structure based on the thermal decomposition of FNWs protected by the mask at high temperatures was observed *in situ* by the HIM.

## Results

### Structure of fullerene nano whiskers

**Figure 1:**
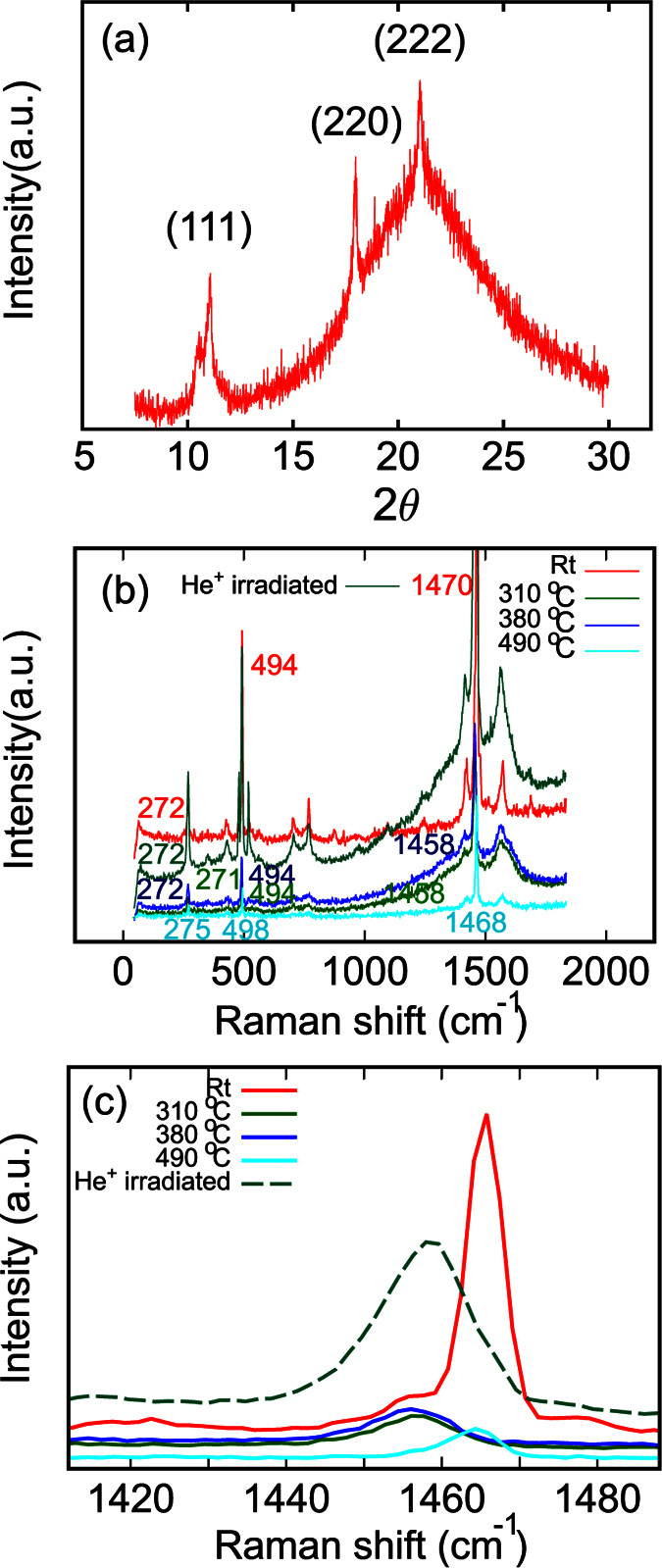
FNWs observed by X-ray diffraction and Raman spectroscopy. (**a**) is the X-ray diffraction results of FNWs annealed at 315 °C for 3 hours. (**b**) is the Raman spectrum of pristine FNWs and FNWs annealed at 310 °C, 380 °C, and 490 °C. (**c**) is the Raman peak of A_g_ mode of FNWs.

The as-grown FNWs synthesized by using a toluene solution of C_60_ and isopropyl alcohol (IPA) with an liquid-liquid interfacial precipitation (LLIP) method had a hexagonal structure with a lattice constant of *a* = 2.405 nm and *b* = 1.001 nm measured by X-ray diffraction (XRD) and high resolution transmission electron microscopy fast Fourier transform (HRTEM-FFT) pattern. After the solvent evaporation, C_60_ FNWs formed with an fcc structure with a cell dimension *a* = 1.423 nm, as indicated by the XRD characterization^27^. The FNWs annealed at 310 °C for 2 h were also checked by XRD as shown in Fig. 1(a). The diffraction result was indexed by an fcc system. This result is consistent with the results obtained by Minato and Miyazawa^27^.

The FNWs annealed at different temperatures or irradiated by 30 keV He^+^ ions were characterized by Raman spectroscopy, as shown in Fig. 1(b). Peaks around 272 cm^−1^, 496 cm^−1^, and 1470 cm^−1^ can be assigned to H_g_ squashing mode, A_g_ breathing mode, and A_g_ tangential stretching mode of fullerene molecules, respectively^28^,^29^. Figure 1(c) shows the details of Raman peaks around 1470 cm^−1^. Red lines in Fig. 1(b,c) are Raman spectra obtained with as prepared FNWs. A high intensity peak at 1470 cm^−1^ and a shoulder peak at 1460 cm^−1^ indicate that parts of the sample were polymerized^28^,^30^. After annealed at 310 °C and 380 °C, the peak at 1470 cm^−1^ disappeared. Peaks at 1460 cm^−1^ show that all the fullerene molecules in FNWs were polymerized. Since all the samples were characterized by Raman with the same power laser, laser induced polymerization is not considered for contribution^30^. The polymerization can be attributed to thermal induced polymerization or sublimation of the pristine fullerene molecules^9^,^10^,^28^. After annealed at 490 °C, the peak at 1460 cm^−1^ shifted to 1468 cm^−1^ again. This result is due to the thermal decomposition of the polymerized FNWs and will be discussed in this paper. Similar to the samples annealed at 310 °C and 380 °C, He^+^ irradiated FNW also shows a single peak at 1460 cm^−1^. The polymerization is attributed to high energy charged particle induce polymerization of C_60_ molecules. This is also observed in TEM observation of C_60_ films and FNWs^10^,^31^.

### Thermal decomposition of fullerene nano whiskers

**Figure 2:**
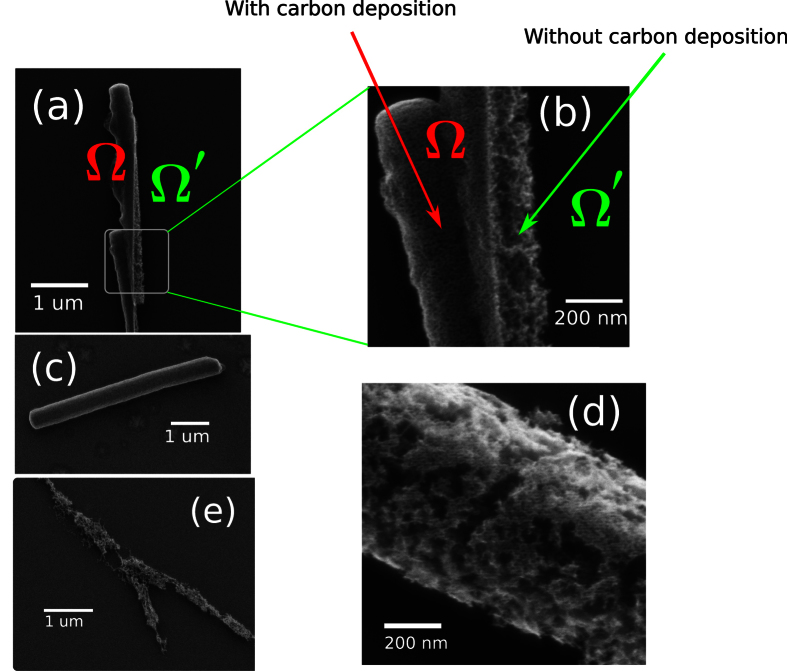
FNWs observed by HIM at 490 °C. (**a**,**b**) are images of a FNW was scanned with different fields. Ω indicates the area that covered by *a*C film which protects the FNW from decomposition. Ω′ indicates the area of decomposed FNW. (**c**) Is FNW continuously scanned from room temperature to 490 °C. (**d**) Is an image of a FNW were scanned at 490 °C after which were scanned at 380 °C. (**e**) Is a FNW scanned at 490 °C once.

Figure 2(a,b) show a special FNW with two different kinds of surface morphology. Two different areas, Ω and Ω′, are clearly separated by a boundary between them. The sample was checked on the surface of the *in situ* heating sample holder for two rounds. In the first round, the FNW was scanned at a temperature lower than 300 °C. The *a*C was deposited on the scan area as indicated by the Ω in Fig. 2(a,b), respectively. After that, the FNW was heated to 490 °C. The sublimation of FNW area covered by the deposited *a*C film was found to be suppressed by the protection effect of the over-layer film. Then, the images shown in Fig. 2(a,b) were obtained with the second round of scan performed at 490 °C.

Figure 2(c–e) show three different typical FNWs with different processes. The FNW in Fig. 2(c) was scanned several rounds from RT to high temperature. In the last round, the image was taken as shown in Fig. 2(c). The fullerene molecules were protected from sublimation by deposited *a*C films from the surface to the center of the whiskers. The FNW in Fig. 2(d) was also scanned twice. The sample was scanned at 380 °C first and then at 490 °C. During heating, the inner C_60_ of the FNW was sublimated by the high temperature and then a fullerene shell generated. The FNW in Fig. 2(e) was observe at 490 °C once and then taken an image. Without protection from deposited *a*C, the FNW in Fig. 2(e) was totally destroyed by sublimation. Only the broken C_60_ residues were left on the substrate.

## Discussion

Hollow structures of FNWs similar to Fig. 2(d) have been observed and published by several groups including our previous works. Xu and Fujita *et al*. found that the FNWs are covered with C_60_ oxide layers in an ambient atmosphere by using scanning Auger microscopy and conductive atomic force microscopy^5^. The different stabilities of the outer C_60_ oxide layer and the inner molecular crystal provide a method of creating a shell structure by sublimating the inner C_60_ crystal. A C_60_ shell structure, called “cocoon”, was first prepared by Haluška *et al*. by heating C_60_ crystal in a vacuum environment^3^. The shell structure of C_60_ was also observed in FNWs by using high resolution transmission electron microscopy (HRTEM) after the FNWs were annealed in a vacuum at 600 °C. The C_60_ crystal was totally sublimated at 600 °C according to the HRTEM images and selected-area electron diffraction pattern^10^. Only the *a*C shell, or “cocoon” was obtained after heating. The decomposition of FNWs was observed *in situ* by non-contact atomic force microscopy (NC-AFM) by Fujita *et al*. The temperature-dependent morphology of a FNW indicates that the FNW was starting to be decomposed and shrank at ~300 °C^5^.

Based on the thermal stability of FNWs, it is possible to make nano structure on the surface of FNWs with high spacial resolution surface patterning by HIM. The spacial resolution of ion-beam-induced *a*C deposition is determined by beam energy, deposition time, scattering of secondary electrons (SEs) in the substrate and as-deposited nanopillars, and so on^25^. Besides the high energy primary beam, the SEs emitted from the substrate, with energies of less than 50 eV, play an important role in the EBID and IBID, because the cross section for the electron impact dissociation of the hydrocarbon molecules peaks at low energies^15^. The spacial resolution of SE scattering is defined by the spot size of the helium ion beam on the sample surface and the trajectory of the helium ions scattered in the sample. In this work, the de Broglie wave length of helium ions with an energy of 30 keV is 0.083 pm, as calculated from 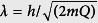, where *h* is Plank’s constant, *m* is the mass of helium ions, and *Q* is the energy of helium ions. The helium ions penetrated the substrate with weak scattering and experienced an electronic loss event. The helium beam was weakly scattered because the ions were lighter than the sample nuclei and much heavier than electrons. This makes the interaction radius between the helium beam and surface of the sample small and the SE yields high^32^. The resolution of the SE images obtained by using HIM was measured by scanning the edge of highly oriented pyrolytic graphite (HOPG) as 0.35 nm.

**Figure 3:**
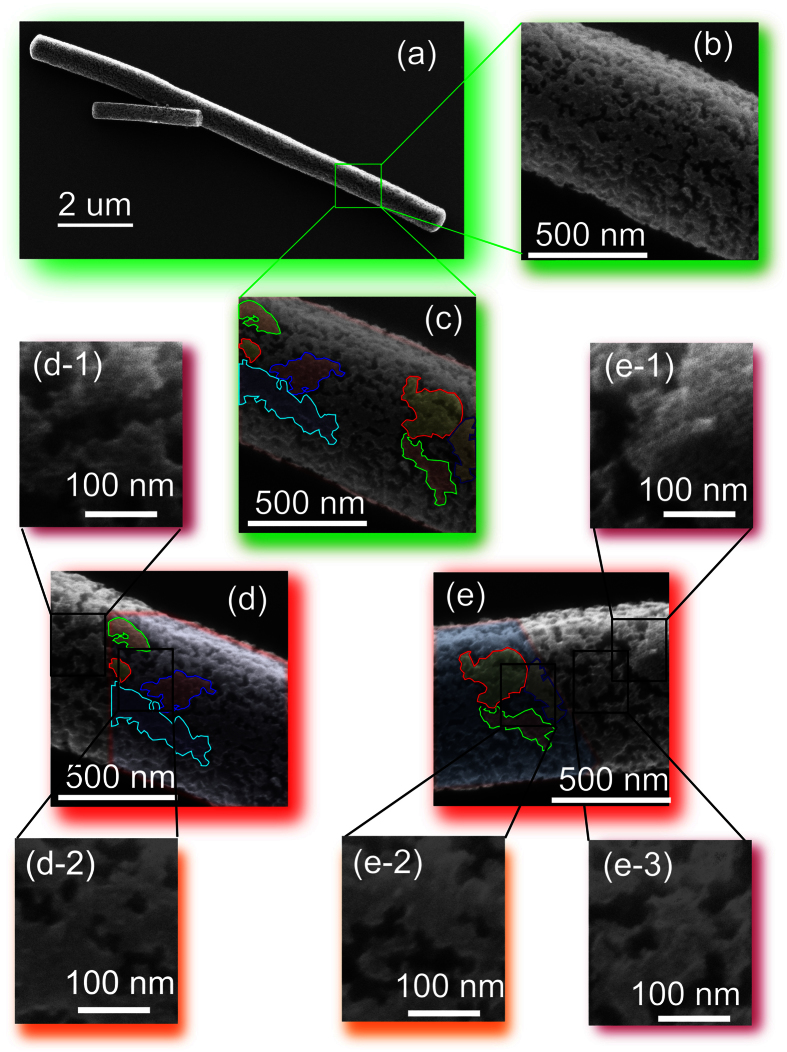
Formation of periodic structure on the surface of the FNW. (**a**–**c**) Are secondary electron images of a FNW scanned at 380 °C. (**b**,**c**) Are secondary electron images of same position as indicated by the green square in (**a**). (**d**,**e**) Are images of FNW scanned at 490 °C. (**d,e**) Are area overlapped with (**c**). Indicative patterns in (**c**) were marked in (**d**,**e**) to show their positions. The details of periodic and non-periodic surface in (**d**,**e**) was shown in (d-1,d-2,e-1,e-2,e-3), respectively.

The periodic structure shown in Fig. 2(d) was made by a sequential scans at 380 °C and 490 °C. At first, the FNW shown in Fig. 3(a) was scanned by a helium ion beam with current of 0.5 pA and dwell time of 12 *μ*s, respectively. Every lines in the image was scanned for 32 times. The size of the image is 1024 × 1024 pixels. Therefore, the interval between every lines is about 12 nm. After that, parts of the FNW shown in Fig. 3(c) was scanned twice to show the details of the FNW morphology. Finally, the sample was kept annealing at 490 °C and scanned to obtain the SE images shown in Fig. 3(b,d,e).

**Figure 4:**
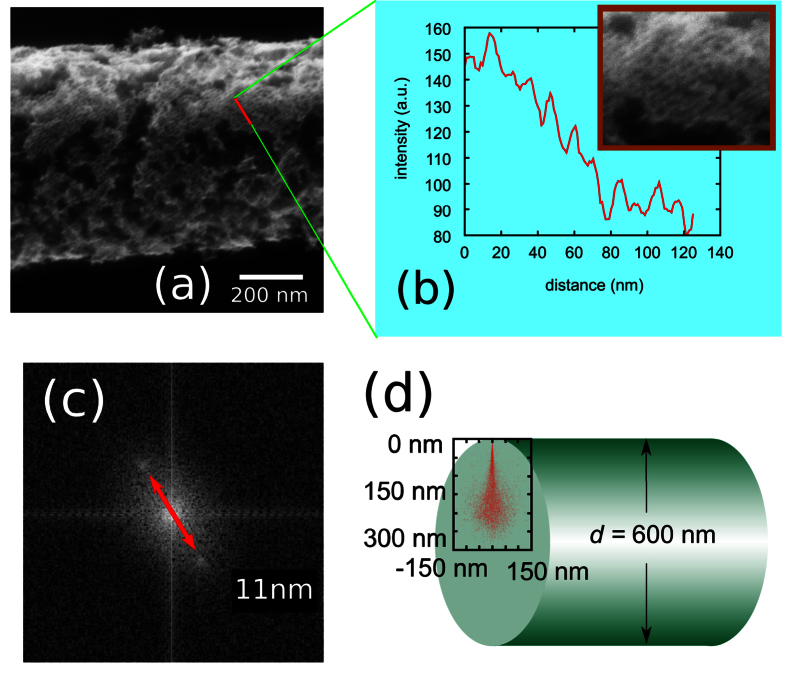
Periodic structure on the surface of FNW scanned at 490 °C after being scanned at 380 °C. (**a**) is secondary electron image obtained by scanning at 30° rotated from the direction of scan in Fig. 2(d). (**b**) is the line profile of periodic structure on the surface of observed FNW. (**c**) has the FFT results for part of (**b**), indicating that period of structure is about 11 nm. (**d**) shows the Monte Carlo simulation of scattering of 30 keV helium ions in FNWs.

During scanning by helium ion beam, amorphous carbon atoms are deposited on the surface by the beam and secondary electrons *via* IBID. The deposited *a*C lines protected the C_60_ molecules from the FNW decomposition. It makes periodic structure on the surface of FNW, as found in the SE images obtained at 490 °C. The periodicity is about 11 nm obtained by the line profile and FFT pattern shown in Fig. 4(b,c), respectively. As shown in Fig. 3(b,c), a part of the FNW was scanned twice at 380 °C with field view of 1 μm. The line intervals in Fig. 3(c) is about 1 nm. Since it was scanned twice with a bit shift, the *a*C lines are overlapped. As a result, *a*C film covered on the surface of FNW homogeneously and protected the FNW from decomposition. Figure 3(d,e) show the morphology of FNW decomposed at 490 °C. We marked 7 indicative patterns in Fig. 3(c) with different color lines. The indicative patterns were marked in (d) and (e) to show the overlapped areas in Fig. 3(c–e,d-2 and e-2) are the overlapped areas in (c) and (d), (e). They show smooth surface since *a*C film is homogeneous on the surface of FNW. (d-1), (e-1), and (e-3) are out of the overlapped area as shown by the indicative patterns. These areas were scanned once to obtain Fig. 3(a). The helium ion beam induced deposited *a*C lines with intervals about 12 nm masked the C_60_ fullerenes from decomposition. Then, periodic structure was formed in these areas as show in Figs 3(d-1and 2(d).

High energy particles irradiation in the surface and bulk of the FNWs induces the polymerization of C_60_ molecules, as we characterized by Raman spectroscopy shown in Fig. 1 10,31. The polymerization was also observed in thermal treated FNWs. However, after annealed at a higher temperature as 490 °C, the polymerized C_60_ molecules decomposed. So, the beam induced polymerization can not form a stable structure on the surface of FNWs. With a Monte Carlo method, we simulated the scattering of He^+^ ions in the FNWs with an energy of 30 keV as shown in Fig. 4(d) 33. The typical diameters of FNWs are various from 300 nm to 1 um as measured by SEM. From the simulation results, we can see that the penetration depth of He^+^ ions in the FNW is about 300 nm. Most of the scattering evens are in a wide range more than 200 nm. In this case, the C_60_ molecules in FNWs are polymerized simultaneously. Therefore, the FNW can not be selectively irradiated by the He^+^ ion beam to form a periodic structure.

Based on the Monte Carlo simulation, we can see that only the surface modification, such as helium ion induced carbon deposition, can form a periodical structure on the surface of FNWs. Since the SEs are important to the EBID and IBID, the resolution of the *a*C deposition is a bit larger than the SE image resolution^15^. With some special techniques, sub 10 nm patterns have been obtained by EBID^17^,^34^,^35^,^36^. Typical resolution of electron beam induced deposition with a 2 nm primary beam is about 15~20 nm measured by experiments and Monte Carlo simulation^37^. Based on its higher secondary electron imaging resolution and surface sensitivity, HIM is advanced in high spacial resolution surface pattering. In Winston’s work, helium ion directly writing lithography with resolution of 20 nm and 10 nm has been introduced^38^.

## Conclusion

In this work, we studied the FNWs at ambient and high temperatures with HIM. It was found that the FNWs can be sublimated at a high temperature when they are exposed to the vacuum environment. The deposition of *a*C on the surface of FNWs protected them from decomposition at a temperatures as high as 490 °C in our *in situ* observations. With Monte Carlo simulation and Raman characterization, we found that helium ion beam induced *a*C film deposition can change the morphology of the FNWs. Based on the high special resolution, an ultra-thin *a*C film deposited on the surface of the FNWs by helium IBID of HIM. The fullerene molecules was selectively protected by the *a*C film from sublimation and form an artificial periodic structure on the surface of annealed FNWs. This experiment confirmed the high spacial resolution of ion beam induced *a*C deposition with HIM.

## Methods

### Fullerene nano whiskers preparation

The FNWs were synthesized by using an LLIP method. A toluene solution saturated with C_60_ was prepared and poured into a glass bottle. Then, isopropyl alcohol was gently added into the bottle to form a liquid-liquid interface between the isopropyl alcohol and the C_60_ -saturated toluene. The bottle was kept at 15 °C. Then, the FNWs were nucleated and grown at the liquid-liquid interface in the glass. After synthesis, the FNWs were transferred onto the Si sheet of the *in situ* heating sample holder from the alcohol solution. After that, they were observed at room (RT) and high temperatures by the HIM.

### Fullerene nano whiskers structure characterization

#### X-ray diffraction

The FNWs were annealed in vacuum with a pressure better than 1.2 × 10^−8^ Torr at 315 °C for 3 h. After that, they were measured by Rigaku RINT powder diffractometer with monochromatized Cu K*α* radiation (*λ* = 0.15405 nm).

#### Raman spectroscopy

The FNWs were annealed in vacuum with pressure better than 1.2 × 10^−8^ Torr at 310 °C, 380 °C, and 490 °C for 30 min. Then, the samples including pristine FNWs were characterized by confocal Raman microscope (Renishaw) with a laser with wavelength of 532 nm. The laser power was 45 mW as calibrated by a power meter. The exposure time was 10 s. The FNWs were also irradiated by helium ion beam with energy of 30 keV, beam current of 5.0 pA, scan area of 1.4 × 1.4 mm^2^, for 5 min and checked by the Raman spectroscopy. Same parameters were applied for Raman characterization.

### Scanning helium ion microscope and *in situ* heating sample holder design

**Figure 5:**
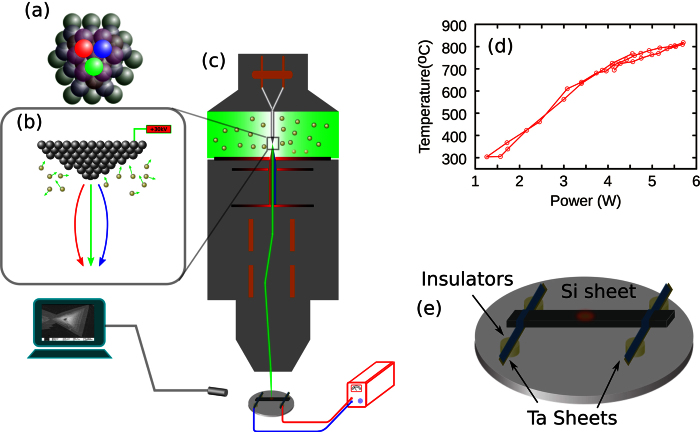
(**a**) Trimer of HIM’s source. (**b**) Source of HIM. (**c**) Structure of HIM. (**d**) The relationship between the temperature of Si sheet and powers applied to it, and (**e**) *in situ* heating sample holder.

The structure of HIM is outlined in Fig. 5(c). A ultra sharp tip was placed in an ultra-high vacuum chamber with only three atoms (trimer) at the apex, called a source, as shown in Fig. 5(a,b). Three helium ion beams were extracted from the trimer and accelerated by acceleration voltage that could be adjusted from 8 keV to 40 keV. Small apertures with diameters of 20 μm, 10 μm, or 5 μm were applied to select one helium ion beam for scanning on the surface of the specimen. An Everhart-Thornley detector was set up in the sample chamber to detect the SEs emitted from the surface of the specimen induced by the inelastic scattering of helium ions and the specimen.

The *in situ* heating sample holder was designed according to the schematic in Fig. 5(e). A silicon (Si) sheet with dimensions of 20 mm × 2 mm × 0.2 mm was fixed onto a copper sample holder by using tantalum (Ta) sheets. The distance between the Ta sheets was 18 mm. The Si sheet was insulated from the copper sample holder by using four ceramics isolating rings with a height of 2 mm. After being baked at temperatures above 300 °C for 72 h, the holder was placed in the sample chamber of the HIM. Two shielded cables connected the Si sheet with a DC power supply to heat the sample. The temperature at the center of the Si sheet was calibrated by the power of the DC current as plotted in Fig. 5(d).

## Additional Information

**How to cite this article**: Guo, H. *et al*. Thermal decomposition of fullerene nanowhiskers protected by amorphous carbon mask. *Sci. Rep.* **6**, 38760; doi: 10.1038/srep38760 (2016).

**Publisher's note:** Springer Nature remains neutral with regard to jurisdictional claims in published maps and institutional affiliations.
